# Phytochemical Study of the Anthelminthic Potential of Guadeloupean Plant Biodiversity

**DOI:** 10.3390/ph17060774

**Published:** 2024-06-13

**Authors:** Tressy Cabald, Carine Marie-Magdeleine, Lucien Philibert, Cédric Caradeuc, Gildas Bertho, Nicolas Giraud, Gerardo Cebrián-Torrejón, Muriel Sylvestre

**Affiliations:** 1 COVACHIM-M2E Laboratory EA 3592, Department of Chemistry, University of the French West Indies, Fouillole Campus, UFR SEN, 97157 Pointe-à-Pitre, Francegerardo.cebrian-torrejon@univ-antilles.fr (G.C.-T.); 2 INRAE, ASSET, 97170 Petit-Bourg, France; lucien.philibert@inra.fr; 3 Laboratory of Pharmacological and Toxicological Chemistry and Biochemistry (UMR 8601 CNRS), University Paris Cité, 75006 Paris, Francegildas.bertho@u-paris.fr (G.B.); nicolas.giraud@parisdescartes.fr (N.G.); 4 BioMedTech Facilities—INSERM US36|CNRS UAR2009, University Paris Cité, 75006 Paris, France

**Keywords:** *Momordica charantia* L., *Carica papaya* L., *Sargassum* spp., anthelmintic, resistances, *Haemonchus contortus* Rudolphi, polyphenols, electrochemistry

## Abstract

Gastrointestinal parasitism is a major health and welfare problem in ruminants. Synthetic chemical anthelmintic drugs have led to the emergence of resistance in gastrointestinal strongyles, inducing the search for alternatives to control the infections that affect ruminants. The objective of this work was to evaluate the anthelmintic potential of plant extracts against *Haemonchus contortus* Rudolphi. Three plants of the Guadeloupean biodiversity, *Momordica charantia* L., *Carica papaya* L. and *Sargassum* spp., were selected based on their high polyphenolic content and natural abundance. The phytochemistry of plants was explored, a biological assay against the parasite *H. contortus* was carried out, and several hypotheses about the way of action were proposed by an innovative electrochemical screening method.

## 1. Introduction

Ruminants in grazing conditions are exposed to several species of parasites such as roundworms, also called strongyles or gastrointestinal nematodes (GIN).

Infections with GIN are the most widespread parasitic diseases. They represent a real obstacle to animal production in the world, particularly in tropical regions [1].

They are a major health problem faced by breeders, leading to significant economic losses due to stunted growth, weight loss and reduced fertility [2].

Among GIN, the barber worm *H. contortus* Rudolphi is considered to be the most pathogenic species that infects sheep [3]. It is a hematophagous parasite that is located mainly in the abomasum of ruminants [4]. It feeds on blood and causes severe anaemia that can lead to the death of severely affected host animals [5].

For more than 50 years, the main control strategy adopted against GIN in sheep and goats has been based mainly on the systematic and repeated use of synthetic anthelmintic (AH) treatments such as thiabendazole, ivermectin and levamisole [6,7,8,9]. The goal is to interrupt the life cycle of the helminths by killing the worms in the hosts in order to reduce the dynamics of infestations [10,11].

However, despite the arsenal of chemical anthelmintics available, the situation is getting worse [12]. Indeed, the intensive and inappropriate use of these drugs has led to the emergence of multi-resistance [13,14].

Moreover, in Guadeloupe (French West Indies), a study showed that all sheep farms in the territory are resistant to benzimidazoles and that the majority of them present resistance to two or three families of synthetic anthelmintics [15].

In addition, these antiparasitic drugs are expensive, and consumers are increasingly sceptical about the use of chemical molecules in farm animals, which could lead to the possible presence of chemical residues in food products and in the environment, thus constituting a pollution risk [16].

Therefore, it is necessary to research and implement alternative or complementary methods of control of gastrointestinal strongyles in order to find and develop new anthelmintic molecules to check and treat parasitic diseases [17].

Different approaches, such as herbal medicine, are used. Medicinal plants contain secondary metabolites that can confer anthelmintic activity [18,19,20,21,22,23].

Polyphenols are secondary metabolites that naturally perform defence functions in plants and could be able to alter the life cycle of gastrointestinal strongyles [11].

The use of biologically active plant extracts rich in polyphenolic compounds, especially tannins, could constitute a future alternative to synthetic anthelmintic molecules [24,25,26,27]. Numerous in vitro and in vivo experiments have demonstrated their anthelmintic effects on the main species of gastrointestinal nematodes, in particular *H. contortus*, thus allowing them to fight against gastrointestinal parasitism in small ruminants [28,29,30,31,32,33].

This study was carried out to assess the in vitro anthelmintic potential of aqueous plant extracts, which could then be easily produced by farmers and added directly to animal feed.

**Figure 1:**
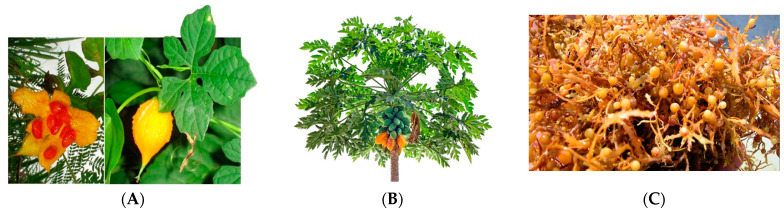
*Momordica charantia* L. (Cucurbitaceae) (**A**); *Carica papaya* L. (Caricaceae) (**B**); *Sargassum* spp. (Sargassaceae) (**C**).

Aqueous extracts were therefore prepared from three selected plants containing polyphenolic molecules: *Momordica charantia* L. (Cucurbitaceae), a pantropical herb whose local vernacular name is Pawoka (Figure 1A); *Carica papaya* L. (Caricaceae) (Figure 1B) and *Sargassum* spp. (Sargassaceae) (Figure 1C). The 3 aqueous extracts were evaluated against infectious L3 larvae of the gastrointestinal parasite *H. contortus* using the larval exsheathment inhibition assay (LEIA).

Our choice was based on previous work concerning the anthelmintic activity of *M. charantia* and *C. papaya* extracts. In the case of *M. charantia*, extracts were tested against *Caenorhabditis elegans* [34], and phenolic compounds (gallic, tannic, caffeic, benzoic and p-coumaric acids, (+)-catechin) and antioxidant activities were reported [35].

Similarly, several studies have explored the anthelmintic potential of *C. papaya* leaves [36]. *C. papaya* leaves also contain rutin, the most abundant flavonoid, and phenolic compounds such as gallic and caffeic acids [37]. In addition, high levels of catechin, naringenin, chlorogenic and syringic acids have been identified in *C. papaya* extracts [38].

Finally, the literature reports that extracts of other *Sargassum* species (*S. fusiforme* (Harv.) Setchell and *S. latifolium* (Agardeh)) have been used as anthelmintics [39,40,41].

In addition, we aimed to develop a reliable, reproducible, fast and innovative technique by electrochemical screening via heme and chitin interaction study in order to rapidly detect extracts or molecules with high AH activity. Our aim is to propose an alternative approach to the various developmental stages of *H. contortus*, such as the egg, with the chitin interaction, and the haematophagous stages, such as L4 worms or adults, with the heme interaction.

## 2. Results and Discussion

### 2.1. Eco Extraction Efficiency

After the eco-extraction step, which combines microwave hydrodiffusion with gravity, allowing the extraction of non-volatile natural substances, good yields were obtained for Pawoka, Papaya and *Sargassum* extracts, respectively 0.86%, 2.31% and 1.58%, in the order of the yields usually obtained at our laboratory with this technique.

This green extraction method offers several advantages compared to conventional extraction techniques usually used, such as a reduction of the extraction time, absence of chemical alteration, high purity extract and high extraction yield.

Alcoholic extracts may be more interesting. However, the aim of the research is to obtain extracts that can be consumed directly by animals and easily produced by farmers. For these reasons, aqueous extracts are more interesting for this study.

### 2.2. Chitin Extraction Yield

The yield obtained for the extraction of chitin is 2.6% (12.8 g). This yield is comparable to other yields obtained in the laboratory for this type of natural polymers.

### 2.3. Quantification of Polyphenols

It is observed that the three extracts active against *H. contortus* (*Sargassum*, Papaya and Pawoka) are rich in polyphenols (Table 1). The concentrations of *Sargassum*, Papaya and Pawoka extracts are 22.125 µg/mL, 153.125 µg/mL and 85.300 µg/mL, respectively. Therefore, it is concluded that the Papaya extract is the most concentrated in polyphenols, while conversely, the *Sargassum* extract is the least concentrated.

### 2.4. NMR-Based Structural Analysis and Metabolomics

The chemical composition of the three plant extracts was determined by ^1^H NMR. For each, it is not absolute concentrations but relative values. In addition to classical 1D NMR experiments, many 2D NMR experiments (^1^H-^1^H COSY, ^1^H-^1^H TOCSY, ^1^H-^1^H NOESY, ^1^H-^13^C HSQC, ^1^H-^13^C HMBC) have been performed to confirm the structure of the chemical compounds found using Chenomx.

**Figure 2:**
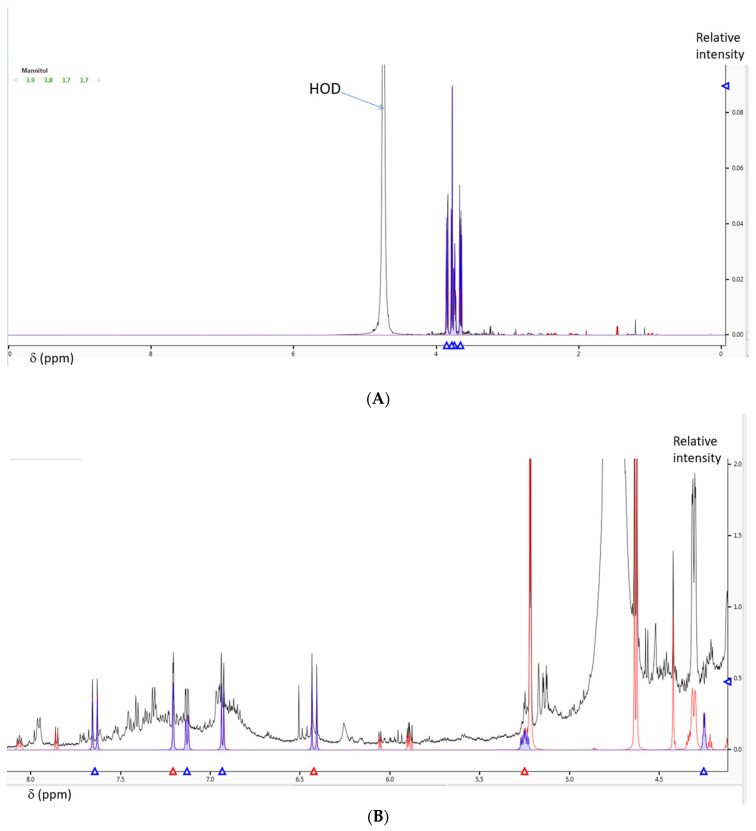

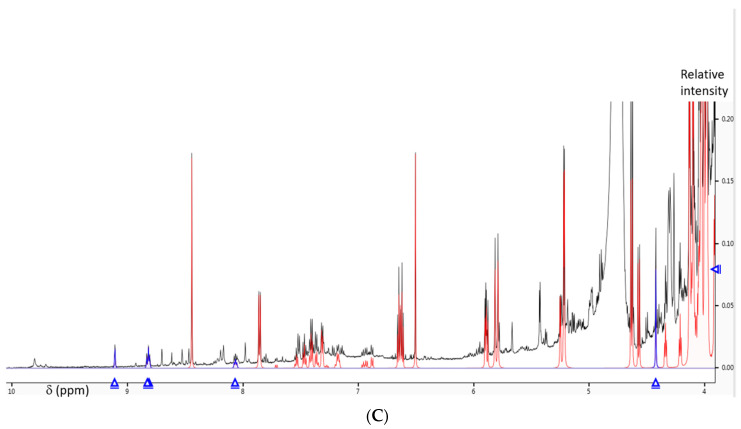
^1^H NMR Spectrum of aqueous extract of *Sargassum*: characterisation of mannitol (**A**); Papaya: characterisation of chlorogenate (**B**); Pawoka: characterisation of trigonelline (**C**).

Not surprisingly, the ^1^H NMR spectrum of the aqueous extract of *Sargassum* shows that it is rich in mannitol but also in amino acids such as alanine, glutamate, glutamine, asparagine and valine (Figure 2A and Table 2A).

The ^1^H NMR analysis of the Papaya aqueous extract allowed the identification and quantification of the most abundant compounds such as glucose, malate, asparagine, alanine, valine 4-aminobutyrate acid or chlorogenate. The phenolic compound chlorogenate, also called 3-O-caffeoyl-D-quinic acid, the alkaloid trigonelline as well as other minor compounds could also be quantified (Figure 2B and Table 2B).

On the ^1^H NMR spectrum of the Papaya extract, in the region between 6 and 9 ppm, numerous signals indicating the presence of several aromatic protons were observed. This finding is related to the proven richness in polyphenols of the Papaya extract previously determined (153.125 µg/mL) (Figure 2B).

The composition of the aqueous extract of Pawoka was determined by metabolomics (Figure 2C and Table 2C). The ^1^H NMR spectrum of this extract shows a high concentration of carboxylic acids (total 15.6686 mM) but also of carbohydrates (total 13.0754 mM) and, to a lesser extent, of amino acids (total 5.5904 mM). We can also note the presence of the alkaloid trigonelline (Figure 2C and Table 2C).

### 2.5. Biological Effects of Extracts

After 70 min, the percentage of exsheathment observed for the negative control (PBS) of each extract was between 94 and 98% (Table 3).

**Figure 3:**
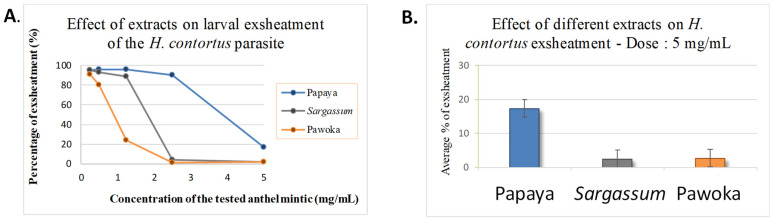
Dose-effect of extracts on larval exsheatment of the *H. contortus* (**A**); Effect of Papaya, *Sargassum* and Pawoka extracts on larval exsheatment of the *H. contortus* parasite at 5 mg/mL concentration (**B**).

Overall, the extracts showed variable average efficiencies, ranging from 7 to 60% (Table 3 and Figure 3). It can be seen that, depending on the concentration tested, the efficiency of Papaya (Figure S2), *Sargassum* (Figure S3) and Pawoka (Figure S4) extracts is not always the same. It is concluded, in relation to the results of the analysis of variance, that there is a significant dose effect for all the extracts (*p* < 0.05).

For Papaya extract, a significant effect was also observed compared to the PBS control (*p* < 0.05). However, the percentages of exsheathment obtained at concentrations of 0.25 mg/mL, 0.5 mg/mL, 1.25 mg/mL and 2.5 mg/mL (Figure 3) did not differ significantly from that obtained for the PBS negative control (*p* > 0.05). It is concluded that at these four concentrations, the extract had no AH activity. We can, therefore, hypothesise that there would be no effective molecules or that they are not in a large enough quantity to allow the inhibition of larval exsheathment in the *H. contortus* parasite.

On the other hand, it can be noticed that the percentage of exsheathment obtained at the concentration of 5 mg/mL (Figure 3) differed significantly from the percentage of exsheathment obtained for the PBS negative control (*p* < 0.05). It is concluded that the extract had AH activity at this concentration. We can, therefore, hypothesise that there are effective molecules for the inhibition of larval exsheathment of the *H. contortus* parasite in the Papaya extract and that they act at a threshold concentration of 5 mg/mL.

For the *Sargassum* extracts, we observed that despite a significant global effect of the extract compared to the PBS control (*p* < 0.05) and a dose effect (*p* < 0.05), the percentage of exsheathment obtained at the concentrations of 0.25 mg/mL and 0.5 mg/mL (Figure 3) did not differ significantly from the percentage of exsheathment obtained for the PBS negative control (*p* > 0.05). It can be concluded that at these two concentrations, the extract had no AH activity. We can, therefore, hypothesise that there were no effective molecules or that they were not in sufficient quantity to allow the larval exsheathment in the *H. contortus* parasite.

However, on the opposite, it can be seen that the percentage of exsheathment obtained at the concentrations of 1.25 mg/mL, 2.5 mg/mL and 5 mg/mL (Figure 3) differed significantly from the percentage of exsheathment obtained for the PBS negative control (*p* > 0.05). It is concluded that at these concentrations, the extract had AH activity. It can, therefore, be hypothesised that there are effective molecules to inhibit larval exsheathment in the *H. contortus* parasite in the *Sargassum* extract at the threshold concentration of 1.25 mg/mL.

For Pawoka extracts, we observed a significant global effect of the extract compared to the PBS control (*p* < 0.05) and a significant dose effect (*p* < 0.05). Moreover, the percentage of exsheathment obtained at the concentration of 0.25 mg/mL (Figure 3) did not differ significantly from the percentage of exsheathment obtained for PBS negative control (*p* > 0.05). It can be concluded that the extract has no AH activity at these concentrations. We can, therefore, hypothesise that there were no effective molecules or that they were not in sufficient quantity to allow the larval exsheathment in the *H. contortus* parasite.

However, on the opposite, it can be seen that the percentage of exsheathment obtained at the concentrations of 1.25 mg/mL, 2.5 mg/mL and 5 mg/mL (Figure 3) differed significantly from the percentage of exsheathment obtained for the PBS negative control (*p* > 0.05). It is concluded that the extract has AH activity at these concentrations. It can be hypothesised that there would be effective molecules in Pawoka extract to inhibit larval exsheathment in the *H. contortus* parasite at a threshold between 0.25 and 0.5 mg/mL.

We observe that at the same maximum concentration (5 mg/mL), the effects of the three effective extracts (Figure 3) were significantly different (between 2.5 and 17.5% inhibition of larval exsheathment; *p* < 0.05), with no significant difference between the *Sargassum* and Pawoka extracts (*p* > 0.05) The latter two extracts, not significantly different, showed a higher efficacy than the Papaya extract of 14.3%.

Furthermore, the threshold effective concentrations observed for the three plant extracts, namely 5 mg/mL for Papaya, 1.25 mg/mL for *Sargassum* and between 0.25 mg/mL and 0.5 mg/mL for Pawoka, demonstrated that the last is the most effective of the three plants (Figure 3). This result is confirmed by the IC50 values calculated, which show the lowest value for Pawoka (Table 3).

### 2.6. Electrochemical Screening

In the study of the interaction with heme, we focused our analysis on the modifications related to the characteristic signal of heme (−0.20 mV). This signal corresponds to the FeIII/FeII reduction (Table 4).

**Figure 4:**
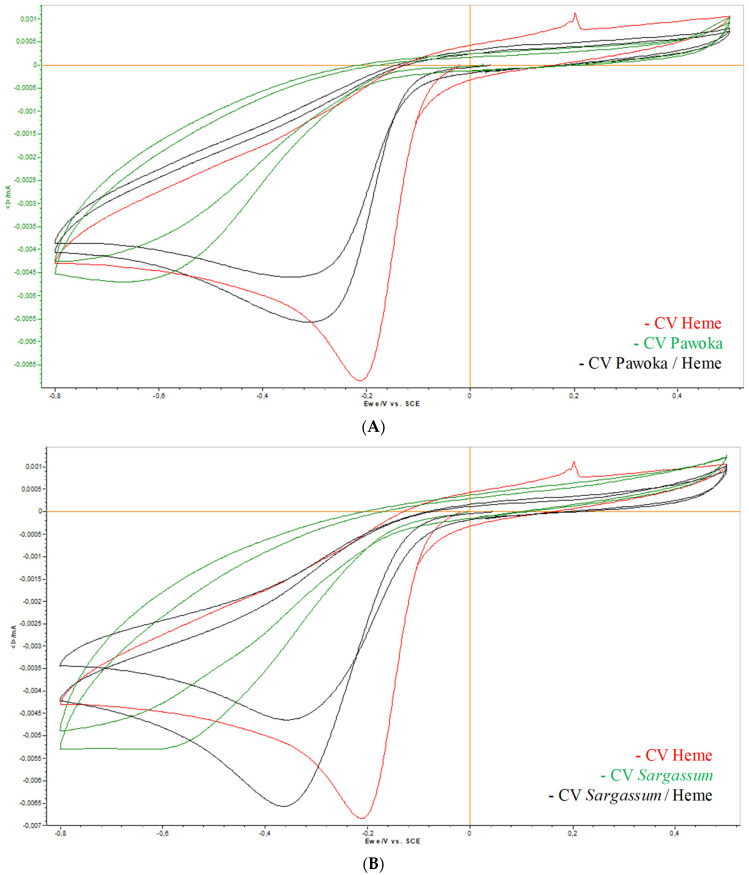

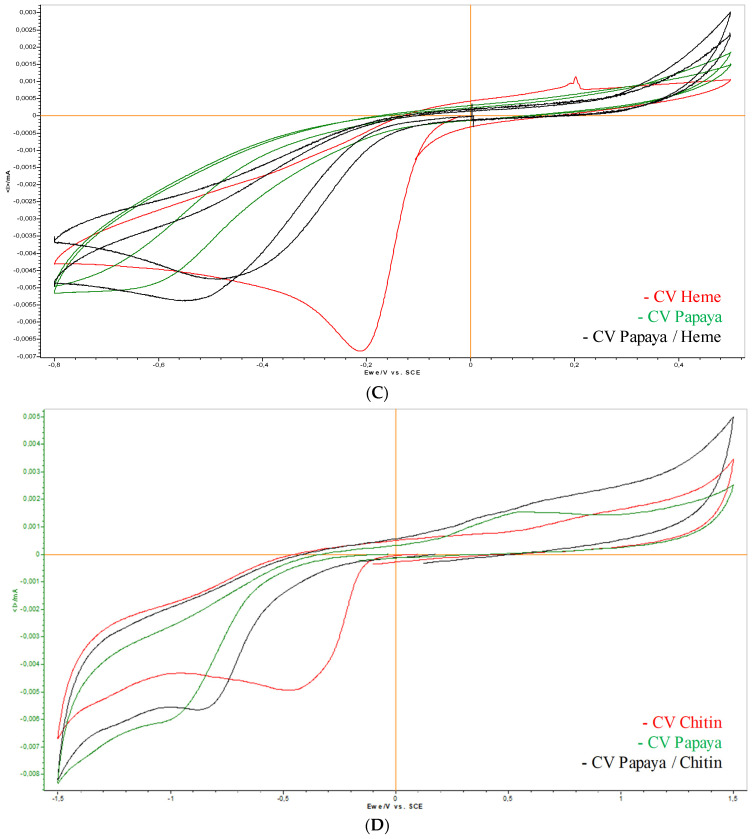

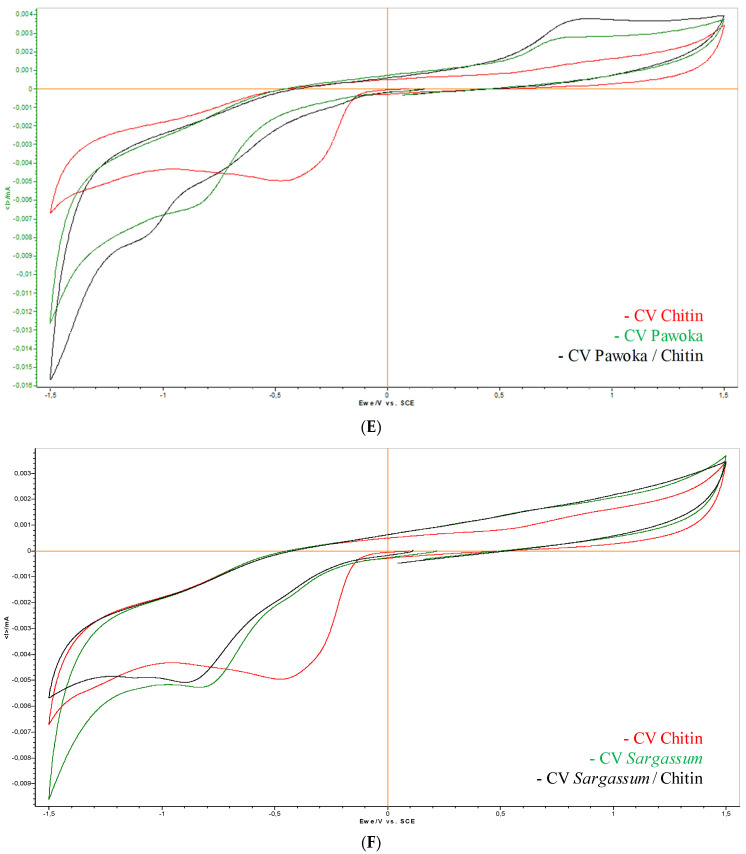
Voltamogram of: Pawoka (heme interaction study) (**A**); *Sargassum* (heme interaction study) (**B**); Papaya extract (heme interaction study) (**C**); Papaya extract (chitin interaction study) (**D**); Pawoka extract (chitin interaction study) (**E**); *Sargassum* extract (chitin interaction study) (**F**).

We observed that the voltammogram (CV) of the active molecule against *H. contortus*, levamisole (Figure S5), and those of Pawoka extract (Figure 4A) and *Sargassum* extract (Figure 4B) each showed a peak almost identical to that recorded for heme, close to the region where the FeIII/FeII reduction occurs. On the other hand, we observed that the voltammogram of thiabendazole (a broad-spectrum AH agent used predominantly in the treatment of intestinal pinworm and strongyloides infection) showed a peak identical to that of heme, located exactly in the region where the reduction of the latter occurs (Figure S6). It is concluded that these three compounds do not bind to heme; therefore, there is no interaction. Another way of action must be involved in the anthelmintic activity of these compounds.

It could be seen that the voltamograms of ivermectin (an endectocidal drug widely used as an anthelmintic in animal husbandry; Figure S7), Papaya extract (Figure 4C), *L. leucocephala* (Lam.) de Wit. (Figure S8) and *M. esculenta* tannins (Figure S9) showed well-defined peaks, which positions were widely shifted with respect to the heme signal. They appeared at more negative potentials than that of heme. We concluded that these compounds bind to heme, so there was an interaction.

In the study of the interaction with chitin, we focused our analysis on the changes concerning the characteristic signal of chitin (−0.40 mV) (Table 5). It was observed that the voltammogram of *L. leucocephala* tannins showed a peak almost identical to that recorded for chitin, close to the region where the reduction occurred (Figure S10). We concluded that these tannins did not bind to chitin therefore there was no interaction.

It can be seen that the voltamograms of ivermectin (Figure S11), levamisole (Figure S12), extracts of Papaya (Figure 4D), Pawoka (Figure 4E), *Sargassum* (Figure 4F), *M. esculenta* tannins (Figure S13), and thiabendazole (Figure S14) show well-defined peaks whose position was very largely shifted from the chitin signal. They appeared at more negative potentials than chitin. We concluded that these compounds bind to chitin, and there was an interaction.

By putting in relation the different results obtained, we observe that our plant extracts all interacted with chitin while only the Papaya extract showed an interaction with heme. It would then be interesting to evaluate the AH activity of our plant extracts at the other stages of development (eggs and L4) of *H contortus*.

## 3. Materials and Methods

### 3.1. Eco Extraction

The fresh leaves of the three selected plants: *M. charantia* L. (Pawoka), *C. papaya* L. (Papaya) and *Sargassum* spp. (a mixture of *Sargsassum natans* (L.) Gaillon and *Sargassum fluitans* (Børgesen) Børgesen), where harvested in Guadeloupe, French West Indies. *Sargassum* was collected on the beach at Saint Felix (Guadeloupe). Plants were identified by the botanist of the University of Antilles (Dr. Alain Rousteau). Plant materials have been sorted. Only healthy, uncontaminated and unfazed leaves were selected. The collected leaves were thoroughly washed with water and air-dried at room temperature. These were stored in airtight bags and kept in the freezer at −20 °C until extraction.

The dried leaf samples were subjected to microwave extraction [42] using the ETHOS X extractor of Milestone-Easycontrol 480 (Sorisole, Italy). The leaves were first weighed, then cut and powdered to facilitate extraction. The plant matrix was then placed in the microwave oven reactor. A volume of distilled water equivalent to the mass was added. The volume of water will change depending on the characteristics of the powder (volume occupied, density, etc.…) when placed in the reactor. The microwave program was adjusted according to the mass of the plant matrix to be extracted (Table S1). The resulting crude products were collected and transferred to containers placed in the freezer until freeze-dried. The freeze-dried dry extracts were transferred to Falcon tubes and stored in the freezer at −20 °C.

### 3.2. Extraction of Chitin

The interaction of anthelmintic compounds with chitin was also explored as it is one of the major compounds of the parasite egg [43].

Lobster carcasses were coarsely cut and then ground into powder. The raw powder was demineralised, 500 g was weighed, and then the flask was placed in a flask containing 650 mL of 2 M HCl. The mixture was heated and held at about 60 °C for 72 h. The demineralised powder was filtered to remove the acid for subsequent deproteinisation; 650 mL of 2 M NaOH was added. The mixture was heated and maintained at 60 °C for 72 h. Finally, the deproteinised powder was filtered to perform discolouration; 650 mL of organic mixture: CHCl_3_/MeOH/H_2_O (1/2/4) was added [44].

### 3.3. Quantification of Total Polyphenols

The quantification of total polyphenols was determined by spectrophotometry using the colourimetric method and the Folin-Ciocalteu reagent [45].

Reagent A (Folin-Ciocalteu) was diluted in a 1:10 ratio with distilled water. This dilution is called RA working solution. To each standard tube, 1.5 mL of reagent C (gallic acid) was added at 5 different concentrations (25, 50, 100, 200 and 300 µg/mL, see Table S2 and Figure S1).

Measurements were carried out on a Microplate Absorbance Reader iMark^TM^ BIO-RAD version V.0, software in End-point analysis protocol (Hercules, CA, USA).

Plant samples were diluted until they reached an absorbance within the limits of the standard curve. Standards were performed in the range of 0 to 300 µg/mL for a volume of 200 µL. 20 μL of each sample (standard and plants) were deposited in a microplate well. 100 μL of working solution and 80 μL of reagent B (7.5% sodium carbonate) were added to each well. Finally, the absorbance was measured at 750 nm. Quantitative analyses of total polyphenols were determined from the linear regression equation of the calibration curve, plotted using gallic acid as the standard (Table S2, Figure S1).

### 3.4. NMR-Based Structural Analysis and Metabolomics

Each sample was prepared in 5 mm NMR tubes using a few milligrams of the extract in 600 µL of D_2_O.

All the samples were measured at 300 K on a Bruker Avance Neo 600 MHz spectrometer (Bruker BioSpin, Wissembourg, France) equipped with a 5 mm BBI probe (Bruker BioSpin, Wissembourg, France) and a SampleJet automation sample changer(Bruker BioSpin, Wissembourg, France).

The spectra were acquired using a classical 1D ^1^H experiment in D_2_O. The data processing was performed using TopSpin 4.0.7. ChenomX NMR Suite 8.6 Professional was used to identify most of the compounds present in the natural extracts.

### 3.5. Biological Assay

The AH activity of the extracts was evaluated by performing the larval exsheathment inhibition assay [46] with a range of concentrations. The objective of the LEIA is to test the anthelmintic efficacy of plant extracts on the inhibition of the exsheathment of infesting *H. contortus* L3 larvae. L3 stage larvae were obtained by recovering and culturing eggs in faeces from animals experimentally infected with *H. contortus* at the animal experimental station of the French National Agricultural Research Institute, Guadeloupe (accreditation to experiment n°A971802). Animals were treated in accordance with the guidelines and regulations for animal experimentation of the French Ministry of Agriculture. The protocol (APAFIS#5527-2016050608133139v2) was validated by the Higher Education and Research under the advice of the Animal Care and Use Committee of French West Indies and Guyana (N°069).

The LEIA test consists of putting in artificial exsheathment of L3 larvae of *H. contortus*, following contact with the extracts to be tested. The effect of the extracts on exsheathment will be demonstrated by a significant inhibition of the latter. Bleach, also known as sodium hypochlorite, has properties similar to the secretions present in the stomach of ruminants, causing the larvae to have an artificial exsheathment. Twenty-one days post-infection, faeces were recovered and cultured for 5 days. The L3 larvae were extracted from the faeces by sedimentation using the Baermann technique [47]. In order to proceed with the test, the previously obtained larval suspension of sheated *H. contortus* was adjusted to a concentration of 1000 L3/mL in phosphate-buffered saline (PBS, pH 7.4; 0.01 M).

Sheathed L3 of *H. contortus* (1 mL) were incubated for 3 h at room temperature with shaking with each of the extracts (1 mL) at different concentrations (5 mg/mL, 2.5 mg/mL, 1.25 mg/mL, 0.5 mg/mL, and 0.25 mg/mL), all of which were diluted in PBS solution. L3 Larvae were also incubated in PBS control. After incubation, the larvae were washed by centrifugation at 2500 rpm for 5 min, three times in PBS. The larvae (100 µL) were then subjected to the process of artificial exsheathment by contact with a solution of 16.6% NaCl and 2.6% active chlorine bleach (1 mL) diluted at 1/140 (dilution required to allow full exsheathment of larvae after 70 min). After 70 min, 200 µL of each tube was removed after homogenisation and transferred into 2 mL Eppendorf tubes, into which 20 µL of lugol was previously dispensed. For each dose of the same extract and each PBS control, five replicates were performed. The contents of each tube were read under a light microscope at ×40 magnification, observing exsheathed and sheathed larvae. The percentage of exsheathed to sheathed larvae is calculated for each replicate of each extract according to the following formula: [Nb L3 exsheathed/(Nb L3 sheathed + Nb L3 exsheathed × 100)].

The obtained data were subjected to variance analysis. The *p*-value of the statistical test, set at 95%, is used to conclude equivalence of means. The concentration required to inhibit the biological response by 50% compared to the control (IC50) was estimated by performing Probit analysis of the dose-response curves of various plant extracts (using Minitab 18 software).

### 3.6. Electrochemical Screening

Cyclic voltammetry (CV) allows the evaluation of the properties of molecules via the detection and the characterisation of electroactive compounds and the study of chemical and electrochemical reaction mechanisms [48]. CV experiments were performed to test a methodology of electrochemical screening of AH properties of molecules and plant extracts via a heme-binding mechanism in an aqueous medium at biological pH [32] and to test the interactions between chitin and the different compounds. For this study, plus the 3 plants evaluated, 3 synthetic AH molecules (levamisole, thiabendazole and ivermectin) and 2 plant extracts (*Leucaena leucocephala* and *Manihot esculenta*) rich in condensed tannins and already known for their AH properties in animals [49,50,51] were assayed. The electrochemical study was carried out using a cell set-up consisting of a three-electrodes system (glassy carbon working electrode, Ag+/AgCl counter electrode and Platinum auxiliary electrode) immersed in the sample solution to be analysed and a potentiostat. A continuous and controlled potential was applied between the reference and the working electrodes. In this study, 10 mg of heme was weighed and then poured into the cell containing 5 mL of phosphate buffer (H_3_PO_4_, pH 7). The cell was put in an ultrasonic bath to homogenise for 5 min. For each sample of plant extracts or AH molecules to be tested, 2 mg was dissolved in ethanol in order to make a microparticle film on the working electrode. The different potentials of heme, sample and heme plus sample were measured. The same protocol was used for chitin [48].

## 4. Conclusions

This study highlights the anthelmintic potential of 3 plants from Guadeloupe’s biodiversity (*M. charantia*, *C. papaya*, *Sargassum* spp.) as an alternative to conventional chemical treatments against *H. contortus*.

The aqueous extracts obtained by eco-extraction, and in particular the *M. charantia* extract, have shown interesting biological activities against this intestinal parasite. These extracts could form part of the daily diet of ruminants. This bioactivity was linked to the quantification of total polyphenols and metabolomic analysis of the extracts.

At the same time, we developed an analytical method for electrochemical screening. This enabled us to explore different routes of action for compounds active against *H. contortus*, such as the interaction of potential anthelmintics with haem (cofactor of haemoglobin) based on the haematophagous nature of the parasite, and the interaction with chitin, a major compound in the parasite’s egg.

This analytical screening, confirmed by biological tests, represents an innovative, reliable and reproducible approach to this major veterinary problem.

In addition, the screening technique developed will be applied to several plants from our rich biodiversity with the aim of carrying out rapid and simple anti-parasite pre-screening. This pre-screening will make it possible to rapidly detect extracts or molecules with high HA potential before launching in vitro trials in order to optimise the time and cost of the experiments.

## Figures and Tables

**Table 1:** Optical densities (OD) of Plant extracts and standard range and concentrations of polyphenols.

Sample	Average OD (750 nm)	Gallic Acid Concentration (µg/mL)
S1	0	0
S2	0.1500	25
S3	0.2615	50
S4	0.3325	100
S5	0.8635	200
S6	1.2100	300
*Sargassum* (1/10)	0.1080	22.125
Papaya (1/10)	0.6320	153.125
Pawoka (1/10)	0.3607	85.300

**Table 2:** Compound characterisation by ^1^H NMR of *Sargassum* sample (**A**); Papaya sample (**B**); and Pawoka sample (**C**).

**A.**	
**Compound Name**	**Relative Concentration (mM) in *Sargassum***
Mannitol	0.3474
Alanine	0.0096
Glutamate	0.0092
Glutamine	0.0090
Aspartate	0.0060
Acetate	0.0026
Valine	0.0024
Lactate	0.0011
Formate	0.0008
**B.**	
**Compound Name**	**Relative Concentration (mM) in Papaya**
Glucose	88.1664
Malate	35.2627
Asparagine	10.2431
Alanine	8.7825
4-aminobutyrate	4.7034
Chlorogenate	4.1628
Valine	2.1308
Trigonelline	2.0848
Leucine	1.9908
Lactate	1.8929
Isoleucine	1.3825
Formate	1.3148
Uridine	0.9758
S-Adenosylhomocysteine	0.7200
**C.**	
**Compounds**	**Relative Concentration (mM) in Pawoka**
Acetate	6.5881
Fructose	5.5754
Glucose	3.6688
2-Octenoate	2.8576
Gluconate	2.3584
Galactose	1.4728
Aspartate	1.3986
Alanine	1.3960
Formate	1.0814
Succinate	1.0653
2-Hydroxybutyrate	1.0585
Propionate	0.9949
4-aminobutyrate	0.9265
Choline	0.8408
Glutamate	0.6515
Valine	0.6485
Methanol	0.6405
Lactate	0.5504
Leucine	0.4479
Asparagine	0.4321
Fumarate	0.4159
Isoleucine	0.3571
Uridine	0.3504
Phenylalanine	0.1766
Trigonelline	0.1533
Benzoate	0.1301
Tyrosine	0.0534
2-Hydroxyphenylacetate	0.0454
Tryptophan	0.0287

**Table 3:** An average percentage of *H. contortus* parasite larval exsheatment was observed following contact of the parasites with the extracts and the control (PBS) and IC50.

	Average Percentage of Exsheatment (%)	IC50 (mg/mL)
Control PBS		
PBS Pawoka	94.04	-
PBS Papaya	97.69	-
PBS *Sargassum*	95.36	-
Plant extracts		
Pawoka	40.21	1.148
Papaya	79.27	4.161
*Sargassum*	57.37	1.980

**Table 4:** Potentials of different plant samples for the study of interaction with heme.

Sample	Potential of Heme (mV)	Potential of Heme + Samples (mV)	Potential of Samples (Control, mV)
Ivermectin	−0.20	−0.72	−0.60
Levamisole	−0.20	−0.16	−0.18
Thiabendazole	−0.20	−0.20	−0.36
Leucene tannins	−0.20	−0.44	−0.42
Manioc tannins	−0.20	−0.40	−0.40
Papaya	−0.20	−0.52	−0.60
Pawoka	−0.20	−0.28	−0.60
*Sargassum*	−0.20	−0.36	−0.56

**Table 5:** Potentials of different samples for the study of interaction with chitin.

Sample	Potential of Chitin (mV)	Potentiel of Chitin + Samples (mV)	Potential of Samples (Control, mV)
Ivermectin	−0.40	−0.70	−0.65
Levamisole	−0.40	−0.65	−0.25
Thiabendazole	−0.40	−0.90	−0.40
Leucene tannins	−0.40	−0.50	−0.80
Manioc tannins	−0.40	−0.75	−0.75
Papaya	−0.40	−0.85	−1
Pawoka	−0.40	−1.10	−0.85
*Sargassum*	−0.40	−0.90	−0.80

## Data Availability

All data are contained within the article and Supplementary Materials.

## Supplementary Materials

The following supporting information can be downloaded at: https://www.mdpi.com/article/10.3390/ph17060774/s1, Figure S1: Calibration curve for the quantification of gallic acid by the Folin-Ciocalteu reagent; Figure S2: Effect of Papaya extract on larval exsheatment of the *H. contortus*; Figure S3: Effect of *Sargassum* extract on larval exsheatment of the *H. contortus*; Figure S4: Effect of Pawoka extract on larval exsheatment of the *H. contortus*; Figure S5: Voltamogram of levimasole (heme interaction study); Figure S6: Voltamogram of thiabendazole (heme interaction study); Figure S7: Voltamogram of ivermectin (heme interaction study); Figure S8: Voltamogram of Leucene tannins (heme interaction study); Figure S9: Voltamogram of Manioc tannins (chitin interaction study); Figure S10: Voltamogram of Leucene tannins (chitin interaction study); Figure S11: Voltamogram of ivermectin (chitin interaction study); Figure S12: Voltamogram of levamisole (chitin interaction study); Figure S13: Voltamogram of Manioc tannins (chitin interaction study); Figure S14: Voltamogram of thiabenzadole (chitin interaction study); Table S1: Microwave program adjusted according to the mass of the plant matrix; Table S2: Preparation of the calibration range.
