# Supplementary material for: Phytochemical Study of the Anthelminthic Potential of Guadeloupean Plant Biodiversity
Source: Pharmaceuticals (Basel). 2024 Jun 13;17(6):774. doi: 10.3390/ph17060774 (PMC11206802; doi:10.3390/ph17060774)
Supplement: Supplementary file 1 [file pharmaceuticals-17-00774-s001.zip › pharmaceuticals-2926266-supplementary.pdf]

Table S1. Microwave program adjusted according to the mass of the plant matrix.

Sample (g Plant/ml H_2O)	Power (W)	Time (min)
Pawoka (130 g/250 ml H_2O)	1580	10
	500	10
	200	40
Papaya (440 g/500 ml H_2O)	1580	10
	500	10
	450	40
Sargassum (1650 g/500 ml H_2O)	1580	10
	1000	10
	500	40
Each program presents a cooling step of 10 min		

Table S2. Preparation of the calibration range.

Sample	Standard (µL)	H_2O (µL)	Gallic acid (µg/mL)
S1 (Blank)	00	200	00
S2	5	195	25
S3	10	190	50
S4	20	180	100
S5	40	160	200
S6	60	140	300

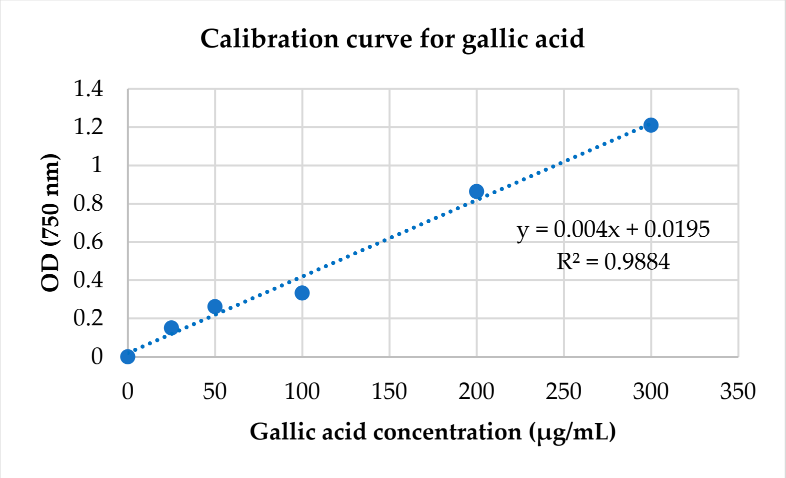

Figure S1. Calibration curve for the quantification of gallic acid by the Folin-Ciocalteu reagent.

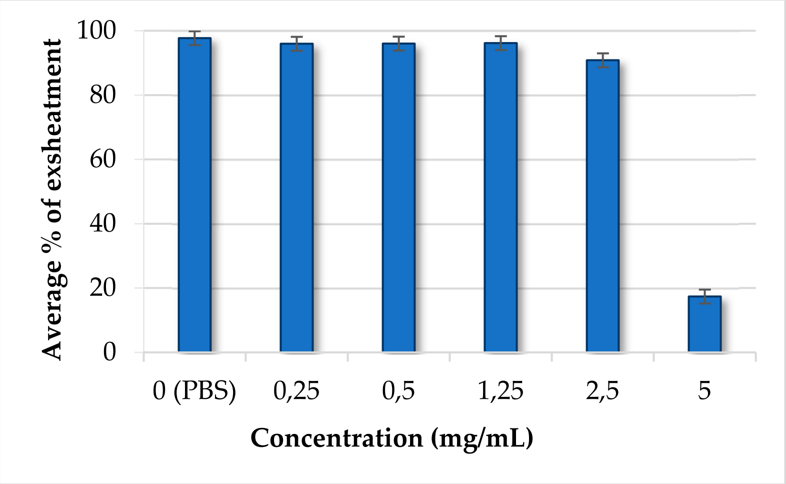

Figure S2. Effect of Papaya extract on larval exsheatment of the *H. contortus*.

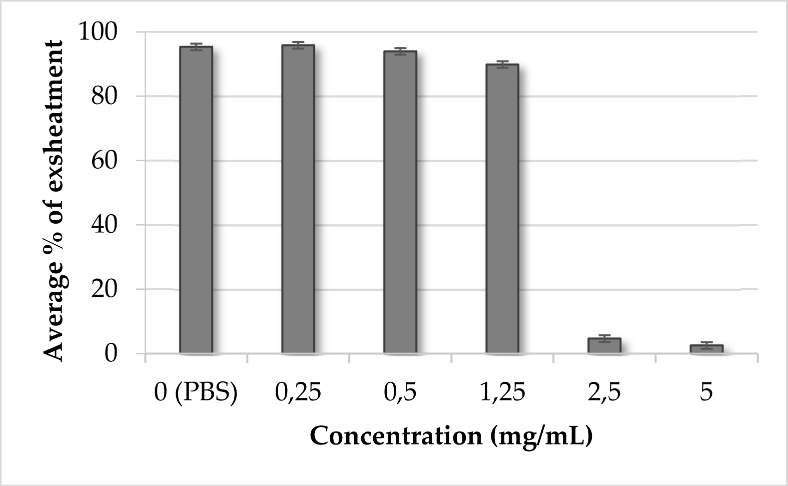

Figure S3. Effect of *Sargassum* extract on larval exsheatment of the *H. contortus*.

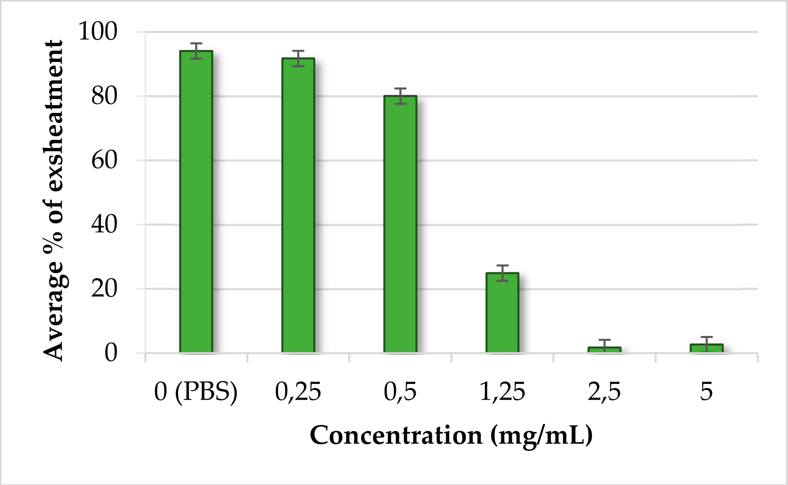

Figure S4. Effect of Pawoka extract on larval exsheatment of the *H. contortus*.

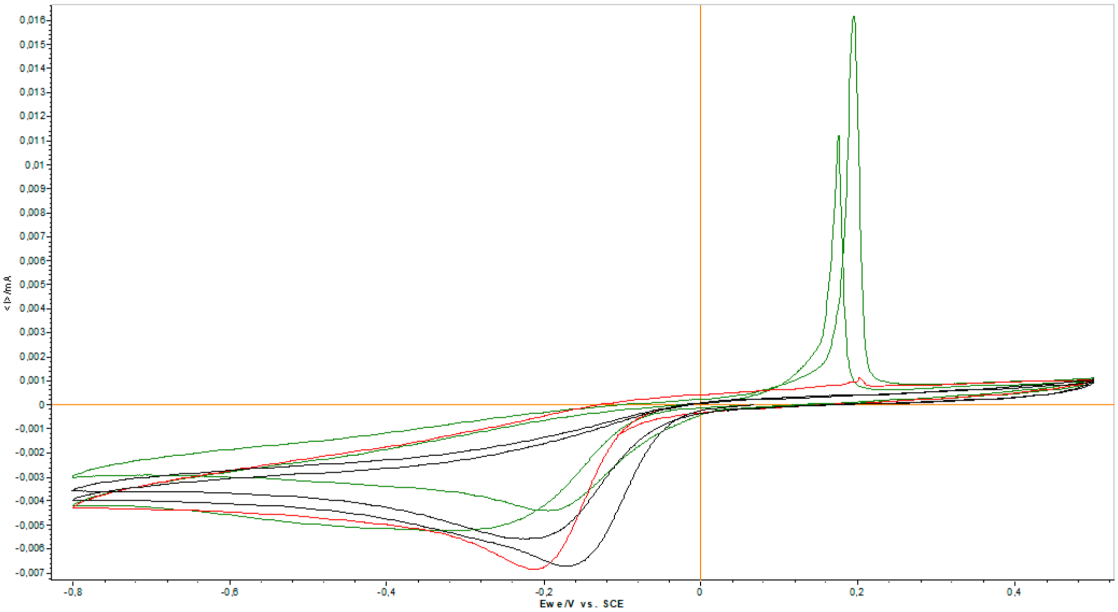

Figure S5. Voltamogram of levimasole (heme interaction study).

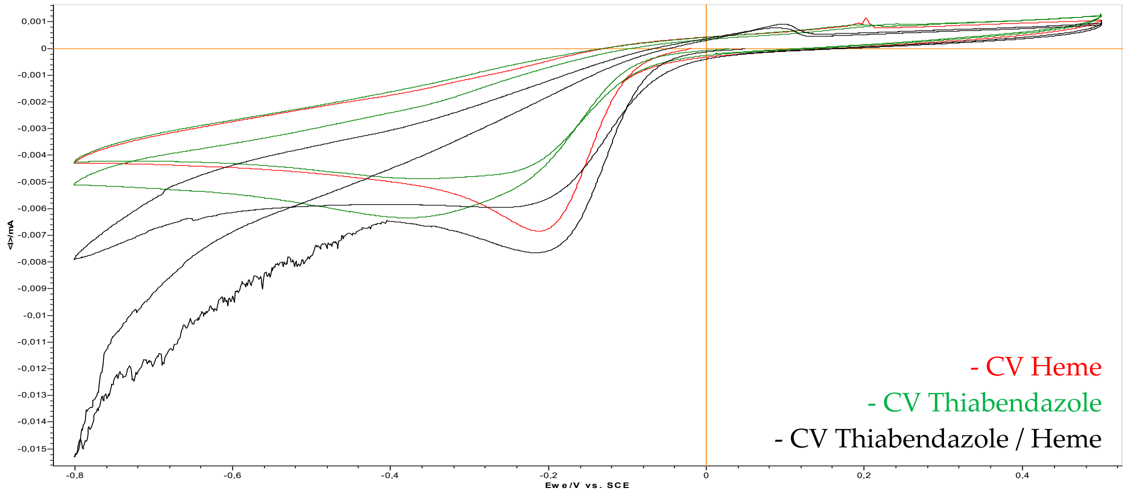

Figure S6. Voltamogram of thiabendazole (heme interaction study).

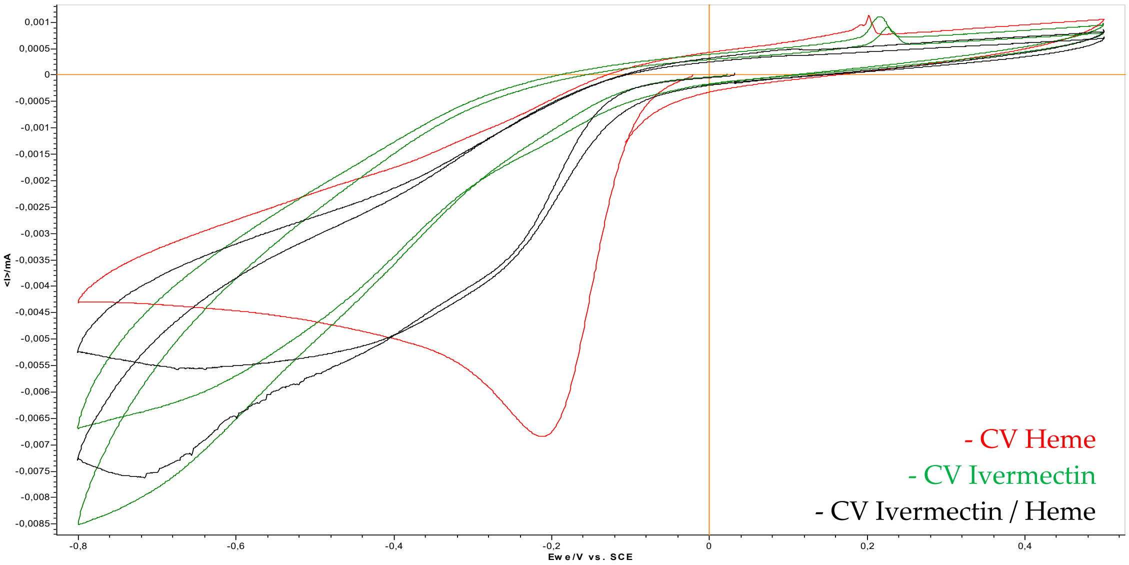

Figure S7. Voltamogram of ivermectin (heme interaction study).

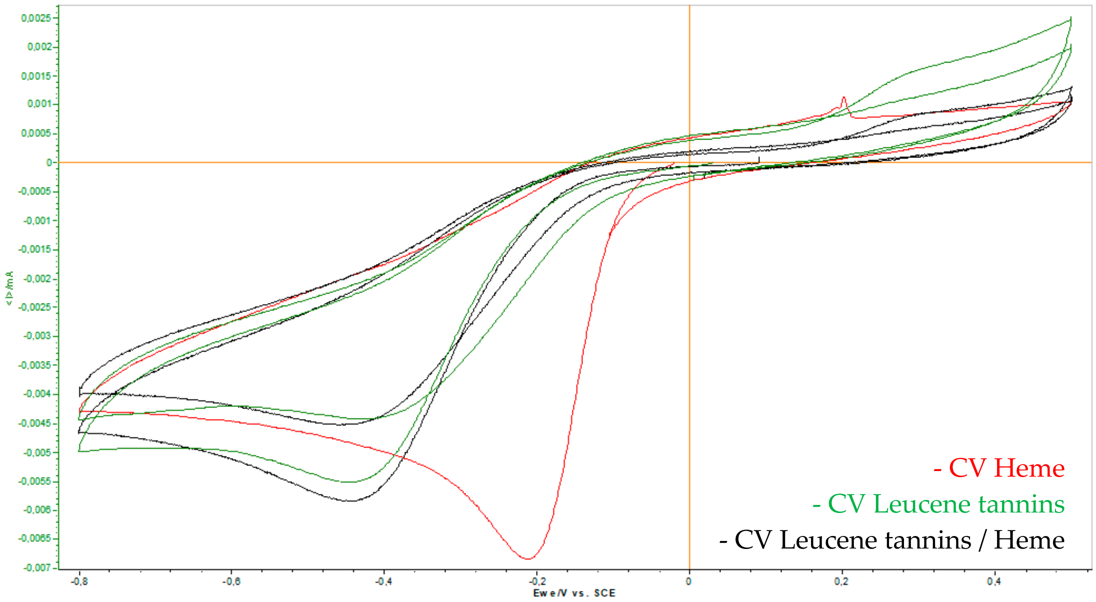

Figure S8. Voltamogram of Leucene tannins (heme interaction study).

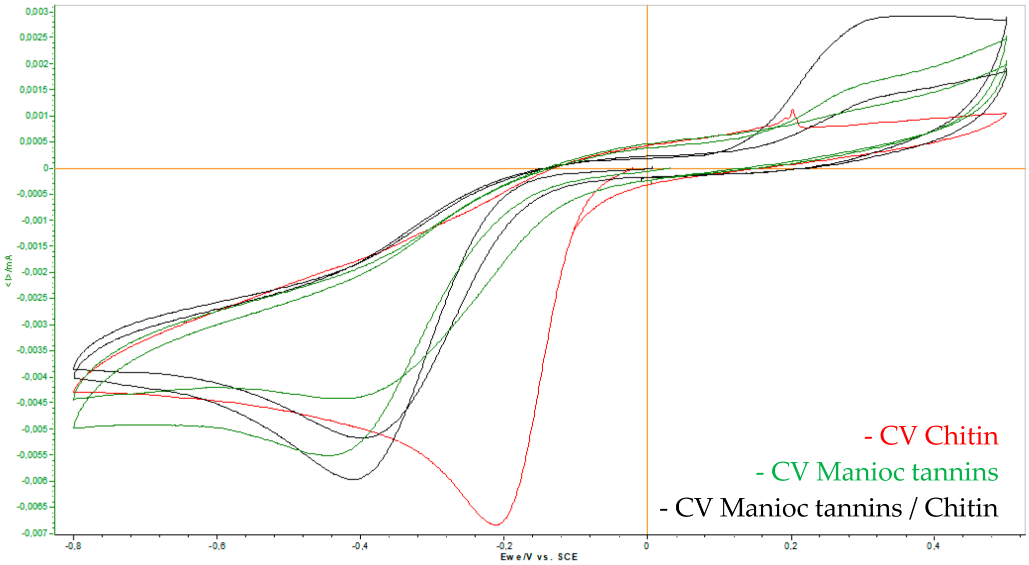

Figure S9. Voltamogram of Manioc tannins (chitin interaction study).

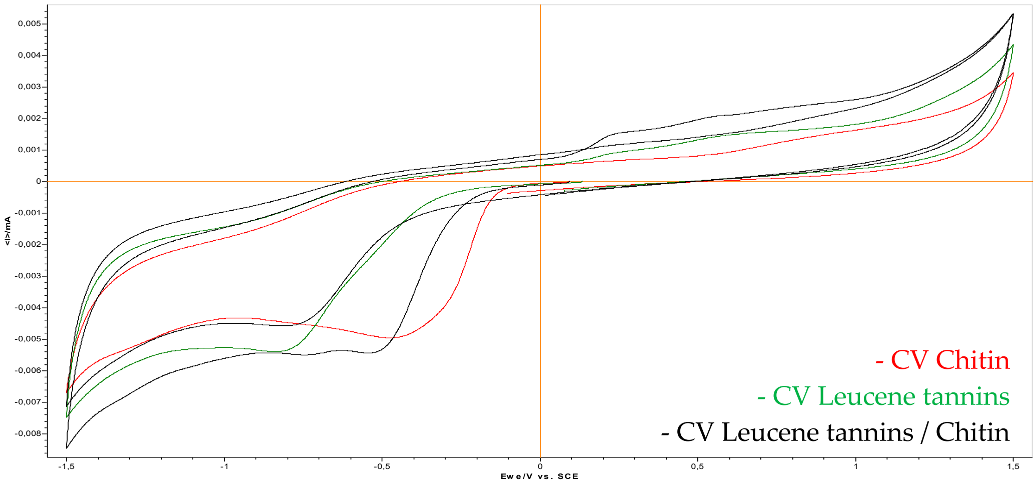

Figure S10. Voltamogram of Leucene tannins (chitin interaction study).

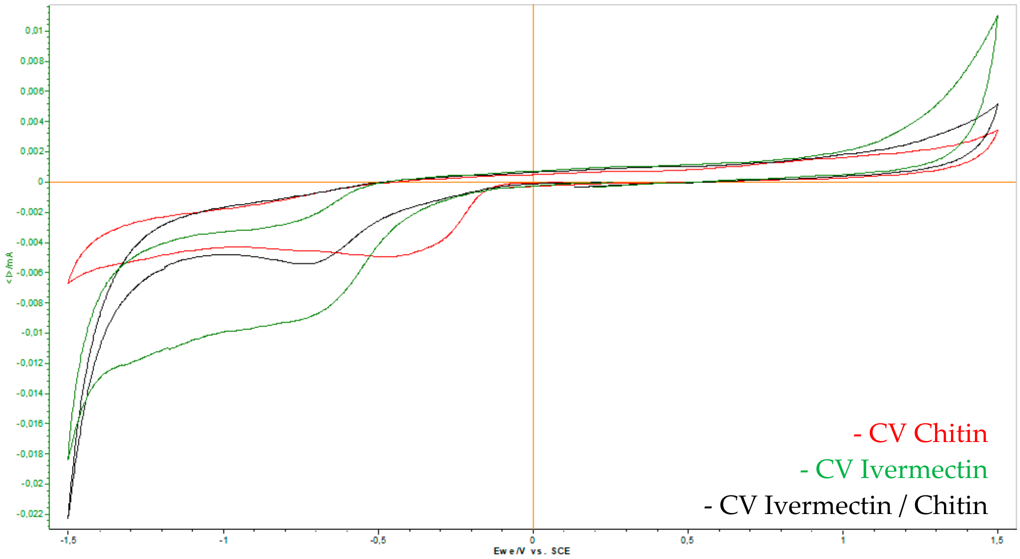

Figure S11. Voltamogram of ivermectin (chitin interaction study).

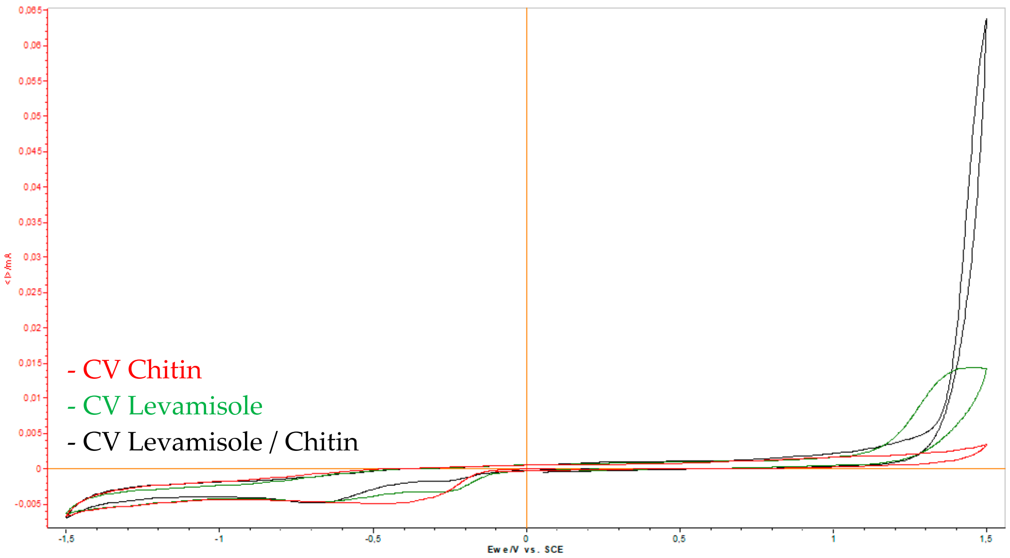

Figure S12. Voltamogram of levamisole (chitin interaction study).

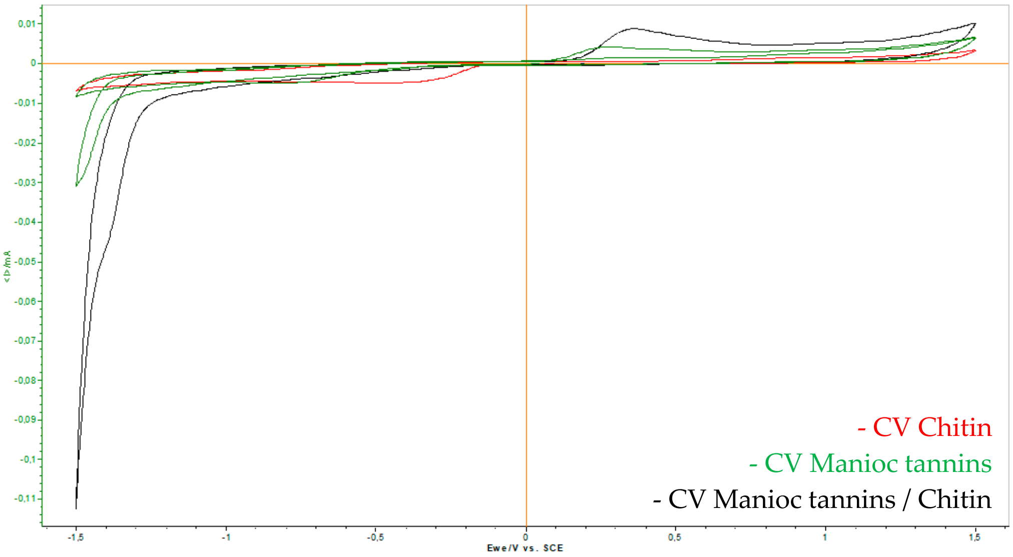

Figure S13. Voltamogram of Manioc tannins (chitin interaction study).

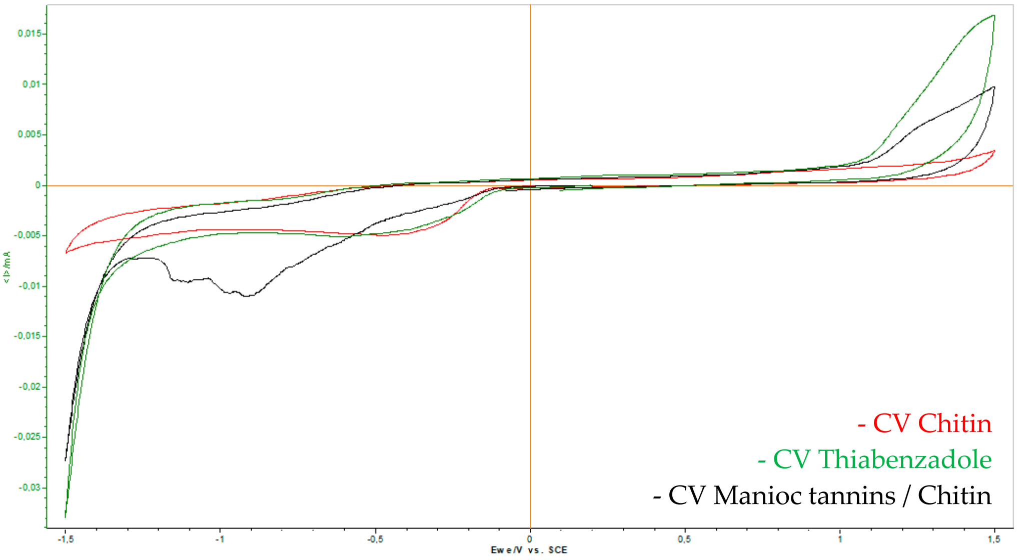

Figure S14. Voltamogram of thiabenzadole (chitin interaction study).
